# A qualitative study of challenges and facilitators to implementing an Indigenous-led cultural safety education program within a large urban emergency department in Vancouver, Canada

**DOI:** 10.1186/s12909-025-07736-0

**Published:** 2025-08-16

**Authors:** Andreas Pilarinos, Jessica Key, Elder Dennis Joseph, Scott Maher, Lori Quinn, Brittany Bingham

**Affiliations:** 1 Indigenous Health Research Unit, Vancouver Coastal Health Research Institute, 6th Floor, 2635 Laurel Street, Vancouver, BC V5Z 1M9 Canada; 2 Providence Health Care, Indigenous Wellness and Reconciliation, 1081 Burrard Street, Vancouver, BC V6Z 1Y6 Canada; 3 Squamish Nation, 320 Seymour Boulevard, North Vancouver, BC V7J 2J3 Canada; 4 Vancouver General Hospital, Emergency Services Program, Vancouver Coastal Health Authority, 920 West 10th Avenue, Vancouver, BC V5Z 1M9 Canada; 5 Faculty of Medicine, University of British Columbia, 2194 Health Sciences Mall, Vancouver, BC V6T 1Z3 Canada

**Keywords:** Indigenous cultural safety, Emergency department, Two-Eyed seeing, Sharing circle methodology

## Abstract

**Background:**

There is growing recognition of the extent and harm of anti-Indigenous racism within public institutions, including healthcare settings. Despite efforts to expand access to Indigenous Cultural Safety (ICS) education programs for healthcare providers, there is limited research examining challenges and facilitators to the implementation of Indigenous-led and -delivered ICS education programs. This study sought to explore the barriers and facilitators to implementing an ICS education program within Western Canada’s largest Emergency Departments.

**Methods:**

This qualitative process evaluation was guided by a Two-Eyed Seeing approach, and the research team included both Indigenous and non-Indigenous researchers committed to conducting culturally safe research. An implementation team consisting of Indigenous and non-Indigenous administrators participated in an Elder-led sharing circle at the mid-point of the targeted delivery of an ICS education pilot program for nurses and allied health professionals in an urban Emergency Department in Vancouver, Canada. The sharing circle was audio recorded and transcribed, and general interpretivist study design and inductive thematic analysis approach were used.

**Results:**

A total of 10 implementation team members, two from the Emergency Department and eight from Indigenous Health Department, participated in a 2-hour sharing circle hosted in May 2022. Several common challenges were identified, including external events and time constraints; program readiness and staff recruitment; and difficulty securing facilitators. Several facilitators to program implementation were also identified, including leader buy-in and cross-departmental communication; on-the-ground project management and coordination; flexible scheduling and accommodations; and facilitator and implementation team support.

**Conclusions:**

These results reinforce the importance of centering Indigenous leadership in the design, implementation, and the evaluation of ICS education programs, while also highlighting the importance of allies in delivering curricula and that facilitators are supported. Efforts are also needed to ensure that programs are flexible and accommodating to address potential recruitment and attendance challenges, including through alternative educational modalities. Lastly, mandating attendance is one approach to addressing recruitment challenges, though compulsory approaches may discourage meaningful engagement.

**Clinical trial registration:**

Not applicable.

**Supplementary Information:**

The online version contains supplementary material available at 10.1186/s12909-025-07736-0.

## Background

Despite the recognized rights of Indigenous Peoples to good health, as stipulated in the United Nations Declaration on the Rights of Indigenous Peoples [1], anti-Indigenous racism persists within healthcare services [2]. This is true in Canada, including in the province of British Columbia, where a recent government commissioned report, titled *In Plain Sight: Addressing Anti-Indigenous Racism Within the BC Health Care* (IPS), documented numerous examples of anti-Indigenous racism within the healthcare system [3], such as through longer wait times for care, inequitable access and a lower likelihood of accessing healthcare, and instances of racism that led to medical malpractice, all of which contribute to high levels of distrust in the healthcare system. These findings have led to several Recommendations and Calls to Action outlining ways to improve cultural safety for Indigenous Peoples within the healthcare system, including through the provision of Indigenous Cultural Safety (ICS) education programs [3–7].

More specifically, ICS education programs provide opportunities to learn about culturally safe healthcare practices and ways to engage Indigenous patients in a respectful and dignifying way, with the ultimate goal of transforming the ways in which healthcare providers engage Indigenous Peoples around their care [3, 8–18]. To date, some research has sought to explore ICS education program implementation and outcomes, outlining important considerations when implementing ICS programs [19–21]. However, existing research notes the persistent absence of Indigenous Peoples’ voices, perspectives, and leaderships in ICS education curriculum development, design, and implementation [19–21]. In light of this knowledge gap, this study sought to explore challenges and facilitators to the implementation of an Indigenous-led ICS education program within Western Canada’s largest Emergency Department (ED).

## Methods

### ‘Hummingbird Level 1’ ICS education program

In recognition of the importance of addressing anti-Indigenous racism throughout healthcare and improving cultural safety, Vancouver Coastal Health has established ICS as one of its core pillars and has committed to providing healthcare staff with relevant educational opportunities. This led to the development of ICS education program, which delivers educational opportunities to healthcare staff. While multiple educational modules are being planned, to date, only the ‘Hummingbird Level 1’ module has been developed and deployed within Vancouver Coastal Health. More specifically, Hummingbird Level 1 includes: (1) an online, self-directed course introducing participants to general ICS concepts; and (2) an interactive, in-person, half-day course that is co-led by an Elder and based on the KAIROS blanket exercise [22, 23]. The Hummingbird Level 1 course is introductory and intended to provide participants with a historical recollection of colonization in Canada, key definitions, as well as foundational knowledge on anti-Indigenous racism in healthcare. For this pilot, the ICS education program was delivered on a weekly basis between March and August 2022, and was specifically intended for ED staff, including nurses, registration clerks, and allied health professionals. ED staff were provided with shift coverage and paid time off to attend the Hummingbird Level 1 ICS education program.

### Study design

This study was led by the Vancouver Coastal Health Research Institute’s Indigenous Health Research Unit, which is an Indigenous-specific, healthcare-embedded research team. For this work, a research team, consisting of Indigenous and non-Indigenous researchers committed to conducting culturally safe research, employed a Two-Eyed Seeing approach. Two-Eyed Seeing is a concept coined by Mi’kmaq Elders Albert and Murdena Marshall [24, 25] and outlines the importance of upholding and respecting both Indigenous Ways of Knowing, Being, and Doing alongside Western epistemology.

Implementation team members were invited to participate in a mid-point sharing circle that was co-led by an Elder and research team members. Implementation team members included both Indigenous Health and ED staff who were involved in the creation and delivery of the ICS education program to nursing staff and allied health professionals who worked within the ED. A semi-structured sharing circle question guide sought to understand challenges to implementing the ICS education program, as well as potential facilitators to its successful implementation, which are detailed in Table 1. This was drafted by the research team and finalized through consultation with the implementation team. The sharing circle was audio recorded and subsequently transcribed into a transcript that was used for analysis.

**Table 1:** Semi-structured Sharing Circle question guide

Questions
1. What have been some of the challenges of the implementation of the ICS program at the emergency department?
2. What was needed to mitigate or address those challenges?
3. What are some foreseeable challenges for the remainder of the project?
4. What is needed to mitigate or address those challenges?

### Analytic approach

Analysis was guided by a general interpretivist study design [26] and inductive thematic analysis was used to explore participants perspectives [27]. This involved developing a preliminary codebook with pre-existing major categories (i.e., barriers and facilitators) as described in Supplementary Material 1, which were then refined through the inclusion of additional codes until themes began to emerge. This continued until we reached saturation, which was determined when we began observing recurring themes and no new insights. All thematic analysis was conducted using NVivo 12 (version 12.7.0, 3873).

## Results

A total of 10 implementation team members, two from the Emergency Department and 8 from Indigenous Health Department, participated in a 2-hour sharing circle to discuss the implementation of the ICS education program. Participants’ roles included ED administrators, ICS team administrators, ICS facilitators, and research team members. This was hosted at the mid-point of the ICS education program pilot rollout in May 2022. Findings were thematically categorized into challenges encountered during the implementation of the ICS education program (Table 2) as well as facilitators to its implementation (Table 3), which are described hereafter.

**Table 2:** Challenges to ICS education program implementation

Theme	Example quote
External events and time constraints	“A global pandemic that put this project on hold for several years, and for good reason. But it did have a significant impact on that planning phase. And that’s because we were held off. It can’t happen, obviously, because the ED [sic] was a hub caring for people during this time. And so as a planning team we were really on hold and then [wondering], ‘Are things getting better? They might be getting a little bit better, we might be ready soon. Oh, nope, there’s another wave! No, we’re not ready. Okay, now we might be ready again. Nope, false alarm!’ So that kind of back-and-forth, and then all of a sudden, ‘We’re ready, let’s go!’”
	“I think challenges definitely were a pandemic. And then we had some other challenges. We had a heat dome, the ongoing opioid crisis, like all that stuff sort of was always in this timeframe. And when the In Plain Sight report came out, there was this real [urgency to] want to learn and do some training.”
	“I think the window of opportunity was really small coming out of COVID. And then having to prepare for [the new electronic medical record system] to go live, which is very resource intensive. So, we had this moment that we needed to use [to deliver the ICS education program], and I think we pretty much did that.”
	*“*Honestly, it doesn’t seem like it was maybe a challenge - but it definitely was – was space for the course. Because that’s the whole thing, and I think that needs to definitely be reflected in the research, that if this is a priority then that space needs to be made. And we got space, because of relationships, because of you, we have a friend.”
Program readiness and staff recruitment	“From the ED [sic] side of things… ICS was a few letters on a whiteboard for a while. [It was] talked about initially, and then gone because of the COVID pandemic, and then brought back, and we were told ‘this what we’re going to do’. And for me, understanding what the ask was, understanding what ICS was and what was needed for the staff to be ready, I didn’t understand that initially and so it was a knowledge gap for me.”
	“From the ED [sic] side of it, it was, ‘Okay, everybody’s going to be paid, and we’re going to do this’. But then it was the figuring out, how we’re going to pay them? It’s involved multiple portfolios [with] different systems, and a lot of navigating that.”
	“Trying to work out how to put this onto an already taxed staff was really difficult. They’re working – it’s already been two years [of crises] – and it’s really difficult [on them]. They have had enough. And so how are we going to put this [program], which is extremely important and coming from a really good place, how do we get our people through it? How do we word that in a way that’s going to get the best buy-in possible?”
	“The challenge is [only delivering the course on] one day, or one Monday, at this time. Some people just always work that shift, so yeah, we get them available, but we weren’t able to backfill people to go [to the ICS session].”
	“And then the only other thing that I don’t have an answer for this is just around that resistance piece. So, how do we know that those who may not be providing culturally safe care, have actually registered for the training? Because most who register are committed and saying “I’m there, and I’m going to do the training, and I’m going to learn new teachings.” But do we know about the ones that have resisted, are they the ones that we should be looking out for?”
Difficulty securing facilitators	“I’ve had experiences where facilitators were saying that they didn’t feel comfortable with delivering some of the slide deck.”
	“One thing I just want to add quickly is that this is a very challenging time in history to be an Indigenous person doing this training. And I just think that that’s something to be mindful of in that other periods of time may not be as challenging. We had [IPS], we had Joyce Echaquan, we had 215, it was literally one thing after another. And with 215, people’s individual communities started doing ground penetrating radar. My community just started ground penetrating radar. It has really challenged people to be able to offer this content and to sit around the circle and talk about it and put yourself out there. So, I think that’s just something for us to be aware of what’s going on in our history and our political ecosystem, and how that affects people right now.”
	“I think that we had challenges with delivery in terms of the facilitators, and so what does that training piece look like to be able to deliver the course? And what that translated into was that it was one or two or three people that can deliver the course and there was a lot of urgency around people having to now drop their commitments to be able to go and deliver the course.”

**Table 3:** Facilitators to ICS education program implementation

Theme	Example quote
Leader buy-in and cross-department communication	“It’s been the most successful if it’s been driven and approved by senior leadership, I think getting buy-in, getting commitments and promotion of this initiative is really beneficial.”
	And it was really tricky for me to understand [what the ask was and what was needed from staff] for a little while until we sat and had a few of these meetings, and then it was really intuitive.”
	“I guess the communications piece is very key because I can appreciate working within a hospital setting, but I can only imagine it’s very stressful…. So that comms [sic] piece is really quite important, to say ‘This is coming, this is what we’re trying to achieve.’”
	“I guess thinking about the planning stage, it was all about the readiness. So, was the Indigenous Health department ready for this? Was the acute site ready for this? Do we have enough resources to roll this out? It was kind a lot of those questions before even thinking about getting this underway.
	*“*For me, to be able to mitigate [the challenges we experienced] was having a really great ED [sic] team. Even though we’re changing a lot, we had a lot of people, they’re a smart team, a really engaged team, who wants to put their hand up to do some work. So, they’re very passionate about cultural safety, mental health, all those sorts of things. I had people that I could wind up [sic] leaning on, and certainly help sell on the education side as well to really help push this. And then as a group, we’ve worked out ‘this is how we can sort of approach it.’”
On-the-ground project management and coordination	“Having one person on this team and one person on the site team that can have their eyes on it from beginning to end, through all of these phases, is really important. Because I think those changes for us created a challenge when we’re trying to be in the middle of rolling something out.”
	“We do rolling talks sometimes in our department where somebody comes from a different area and might do a morning information session where they roll around and talk about the programs we’re doing, the dates that they will be available, and how to sign up on the board.”
	“And then the project manager piece with like timelines, and having that really organized, like keeping us focused and together. Oh, I know, the other challenge was, we had to consider CST-Cerner [sic] rollout. So that timeframe window we put in, it was do it now in this time or we delay it again.”
	“Particularly in terms of recruitment, I don’t know if that’s planning or where that sort of sits, but I think that would have been a bit more helpful [to have active participation on the floor], and maybe that would have come with a project manager on the side? I’m not sure. Or is that like us leading it? I don’t know what that looks like, but we heavily weighted on [Emergency Department administrators] to do that work, which is hard because you’re juggling all the other things.”
Flexible scheduling and accommodations	“So I worry that at a smaller site, that you might have lower numbers already, you would really want to over-book almost. Because we did have every class, there were a couple people that would email and cancel 24 h ahead of time.”
	“I don’t know if actually running the training a later day in the week, so there’s like business days ahead of that. With people cancelling the last minute and things like that, [it’s because they] are not completely prepared. It would have been [better if we] had the resources to adapt to that, or to help support people to be prepared to be in the training as well.”
	“I think the timing of the time and dates of the course need more variability. And just thinking about this stuff, a lot of nurses are mothers, and is it possible even have an evening session, which would free their infant childcare along with their days off? Free people to go a little bit more, but just getting that sweet formula, I think, for attendance.”
	“I’ve been to some other trainings that we’ve had for senior leadership and different groups that were planed specifically for that group and we’ve always shared a meal, and that’s been really important. So the whole circle happens, and the training happens, and then we share a meal, and it’s kind of like the final stamp on the circle. We move away from the circle, and we sit, share a meal together, and people get to kind of unwind from what they learned and what they absorbed. And if they were emotional, they get to kind of come down to that next level. And you get to know people more personally, and that’s what I felt was maybe missing at this workshop.”
Facilitator and implementation team support	“I think a lot more needs to go into supporting, nurturing, and really getting a great group of ICS facilitators who are ready to be able to deliver. I think and how resources and the supports are put in place too. Then there’s some change and some direction too, and a little mentoring.”
	“I think another thing that was nice, as a facilitator, when the workshops where here, the facilitators could do their preparation discussions in a separate room before and then debrief after in that room as well. Doesn’t matter so much for the debrief at [the hospital], because everyone was gone. But people were coming in [while we were debriefing] and so sometimes having those discussions was awkward.”
	“I think on our end, we’re working through that, the strategy plans, but that is definitely a challenge. We have now offered a facilitation course for new facilitators. I think the challenge is that this is delivered by Indigenous Health people and so it’ll be good to move towards developing allies in the delivery.”
	“I really like that there are many opportunities throughout this process to share learnings and to share feedback about how we can improve for future work. So I do think that it’s nice that has been woven in so well, throughout the entire process.”
	“I’m also just thinking, how great is it that we get to reflect on this, in this way? Because I don’t think many interventions actually take the time to do this.”

### ICS education program implementation challenges

Participants described several challenges they encountered during the implementation of the ICS education program. These included: external events and time constraints; program readiness and staff recruitment challenges; and difficulty securing ICS facilitators.

### External events and time constraints

Many participants discussed the challenges they experienced in the context of other pressing public health emergencies, including the COVID-19 pandemic, the toxic drug poisoning crisis, and a concurrent climatic emergency or heat dome. These events contributed to delays and uncertainty around the launch of the ICS education program as the ED was inundated with the fallout of these crises. As a result, participants explained how the changing environment and contextual factors contributed to uncertainty around when the ICS education program would commence.

Furthermore, the release of IPS added urgency to the need for ICS education programs across the health authority. Efforts were especially targeted at the ED given IPS was commissioned in response to allegations of a blood alcohol content guessing game targeted at Indigenous patients being played in numerous EDs across BC. As one participant shared, this gave healthcare administrators the impetus, and urgency, to implement the ICS education program, though this came with added pressure. Additionally, Vancouver Coastal Health had simultaneously been planning to implement a new, centralized electronic medical record (EMR) system in the ED in September 2022, following the completion of the ICS education program roll-out. This added pressure on the implementation team who were provided with a small window of opportunity to roll-out the ICS education program.

Lastly, participants also shared that securing space within the hospital was also challenging. Participants explained that being able to access a large enough space within an acute healthcare setting in the middle of the COVID-19 pandemic and other public health crises was difficult. As one participant recollected, it was because of existing networks that the implementation team was able to secure consistent access to space, though future efforts to expand ICS education programs should consider this.

### Program readiness and staff recruitment

A common theme reported by participants was the challenge of preparing the target program and staff, in this case within the ED, to “understand how important this program is and how it can be beneficial”. Participants who were unfamiliar with the ICS education program and its intent expressed their questions when asked to support the delivery of the program. As one participant shared, this knowledge gap led to some confusion, which was identified as something that could hamper ICS education program expansion into other target programs or units.

Similarly, participants described the need for a better understanding of the logistical considerations, ranging from staff registration to compensation. The delivery of the ICS education program involved multiple departments that required coordination support to liaise between teams. This can be seen through one participants’ recollection of working to understand reimbursement processes at the onset of the project, where they described some of the difficult system navigation that was required to ensure ED staff were paid for their time.

Moreover, participants discussed the recruitment challenges they experienced in rolling-out the ICS education program to ED staff. Some participants speculated that lower-than-expected turnout was associated with the ICS education program being delivered “outside of work hours”. As one participant explained, this resulted in the program being viewed as “one extra thing that people decide they don’t have the capacity for”, leading many staff to skip attending or suddenly cancel. Although Monday mornings were identified as the ideal time to host the ICS education program sessions, some participants believed that this timing may have introduced challenges to engaging staff who were otherwise unavailable on Mondays, as shift coverage may not have been the only challenge to their participation (e.g., childcare access).

Another participant expressed concerns that the ICS education program may not be reaching staff who may benefit from participation the most. Given that ICS education program attendance was voluntary, ED staff who were resistant to ICS may have been less likely to register. To some participants, this challenged the futility of the ICS education program because, if true, it meant they would be missing the “1% [of staff] that we get complaints from.”

### Difficulty securing facilitators

Another significant challenge to the delivery of the ICS education program was securing ICS facilitators. Given the ICS education program curriculum contained triggering content, many Indigenous staff found it challenging to deliver the course content. This was especially true for staff that were new to facilitating, who expressed their unpreparedness and discomfort in delivering the slides, subsequently withdrawing as ICS facilitators. Furthermore, the initial discovery of 215 unmarked graves at the former Kamloops Indian Residential School and ongoing discoveries at Indian Residential Schools and other sites across Canada, as well as ongoing examples of Indigenous-specific racism in the healthcare system, compounded existing challenges experienced by Indigenous staff. One participant shared their concerns on the burden on Indigenous staff asked to facilitate ICS education program sessions, and how this had the potential to cause harm.

Given the difficulty in securing ICS facilitators, certain staff were regularly called upon to facilitate ICS sessions due to their experience. This required some staff to shift their attention to supporting the delivery of the ICS education program, which one participant explained took staff away from other roles and responsibilities. As a result, questions arose around the need of an ICS facilitator training program, which was seen as a critical component to the effective delivery of the ICS education program.

### ICS education program implementation facilitators

Despite the challenges that participants shared around the implementation of the ICS education program, they also shared and discussed several factors that facilitated its implementation. These included: leader buy-in and cross-departmental communication; on-the-ground project management and coordination; flexible scheduling and accommodations; and facilitator and implementation team support.

### Leader buy-in and cross-departmental communication

Ensuring senior leaders were supportive of the ICS education program was seen as a critical aspect to its successful implementation. More specifically, participants shared how receiving a directive to support the delivery of ICS education program emphasized its importance and their responsibility in ensuring its successful implementation. Although this was associated with some initial confusion, particularly around what was needed by the ED to support ICS program implementation, the ability to convene teams and discuss the purpose and objectives of the ICS education program was seen as meaningful and fruitful. As one participant shared, this highlighted the importance of developing strong relationships between team members, communicating openly and regularly, and troubleshooting on how to notify, prepare, and motivate staff to attend the ICS education program.

Another factor that was identified as a facilitator to program implementation success was the readiness of implementation team members and the target program to engage in this work. This collective readiness was seen as an important component to establishing a collaborative, solution-oriented team that was confident in its ability to respond to ongoing and new challenges. Recognizing this, one participant commended the implementation team for creating a space for safe, constructive dialogue that was critical to “making it a successful project”.

### On-the-ground project management and coordination

Participants discussed the potential benefits of having on-the-ground project managers or coordinators who could “operationally help with” the delivery of the ICS education program. These people could support program implementation, address staff’s questions and queries, and work to educate, notify, and recruit staff to upcoming ICS education program sessions. As one participant discussed, having a shared point-person between teams may have addressed some of the logistical challenges that were experienced throughout program implementation, while another participant added that they could have also supported staff recruitment.

While the implementation team did have external project coordination support that was facilitated through the VCH Indigenous Health Department, participants described how additional project management or coordination support could have been helpful in meeting the given timelines. In light of the impending EMR training, as a result of the organization’s shift to a centralized system, the added time constraints made it difficult to ensure the ICS education program reached all ED staff. Moreover, the absence of project management or coordination support on the ED side of things resulted in the downloading of administrative tasks to the ED team, increasing their workload and posing challenges to recruitment efforts given limited human resources.

### Flexible scheduling and accommodations

As ICS education program attendance was voluntary, recruitment of ED staff and managing sudden cancellations were an ongoing challenge for the implementation team. Given the added stress that many ED staff were experiencing in the midst of multiple public health emergencies, there were concerns that lower-than-expected-turnout at each session could negatively impact staff’s educational experience and learning outcomes. One participant expressed concerns on the impact this could have on smaller sites, especially in rural or remote settings, suggesting the need to overbook in anticipation of cancellations.

However, other participants attributed attendance challenges and sudden cancellations to staff unpreparedness. In turn, one participant reemphasized the potential benefits of on-the-ground project managers or coordinators who could engage ED staff about upcoming ICS education program sessions and support them to feel prepared. Building on this, another participant hypothesized that attendance challenges and cancellations could be due to familial or other responsibilities, leading to consideration on the potential benefits of providing sessions outside of traditional work hours, or providing childcare. Additionally, sharing a meal was something that was discussed, which aligns with Indigenous Ways of Knowing, Being and Doing.

While refreshments were provided to attendees, one participant explained that sharing a hot meal allows people to discuss what they have learned and an opportunity to debrief in a relational way.

### Facilitator and implementation team support

Ensuring ICS facilitators were supported to deliver ICS education was something that was shared by several participants. This included ensuring that prospective facilitators had ample training and mentorship opportunities, and that safeguards were in place to prevent any potential harms associated with delivering the course content. Adding to this, participants who were both implementation team members and facilitators shared how opportunities to debrief and gather as a team were beneficial to their learning and well-being, but that protected space was needed to ensure that this could happen safely. Nevertheless, participants shared their concerns on the impacts of the ICS education program on Indigenous facilitators, leading one participant to call for more non-Indigenous staff in facilitation roles.

Participants also commented on how the present sharing circle that was being hosted as part of this research study, as well as the bi-weekly implementation team meetings, were important to ensuring implementation team members felt supported. To some participants, this presented a unique opportunity to share the challenges they had experienced while also identifying ways to mitigate them. As one participant shared, they had not previously had the opportunity to engage in sharing circles and reflect on common learnings and solutions, which they found valuable and meaningful.

## Discussion

Findings from the mid-project sharing circle conducted with the ICS implementation team provided insight into the challenges and facilitators to implementing an ICS education program. When discussing ongoing and expected challenges, participants commented on the impacts of external events and time constraints, program readiness and staff recruitment challenges, and the difficulty in securing ICS facilitators. However, participants also pointed to ways to mitigate potential challenges, including through leader buy-in and cross-department communication, project management or coordination support, flexible scheduling and accommodations, and ensuring facilitators and the implementation team are supported.

To ensure that ICS education programs promote cultural safety and do not perpetuate anti-Indigenous racism or colonialism, existing research emphasizes the importance of centering Indigenous Peoples’ voices throughout all stages of ICS curriculum development and implementation [13, 17, 21, 28–30]. As such, one of the key successes of this ICS education program was that it was being led by and for Indigenous Peoples, including during curriculum development, implementation, and evaluation. Furthermore, an Elder from a local First Nation who has experience in ICS work was present to guide conversations and share knowledge throughout the ICS education program, an aspect that many participants expressed their gratitude for. Research suggests that the presence of Elders during ICS education program is meaningful to participants and aids in their understanding of Indigenous Peoples’ and the importance of cultural safety in creating trust and supporting well-being [15, 16, 28, 31].

While acknowledging the importance of having Elders present during ICS education programs, it is equally important to understand the emotional burden that such training can have on Elders, staff and facilitators, especially for those who are Indigenous. To date, evidence suggests that the Indigenous staff’s delegation of responsibilities deemed ‘cultural’ or ‘Indigenous’ is a significant predictor of burnout and attrition due to the associated emotional labour [32], and so participants highlighted the role of non-Indigenous people in facilitating ICS education programs and supporting curriculum delivery. For example, implementation team members discussed the potential for recruiting non-Indigenous people as ICS facilitators to reduce the burden on Indigenous staff, further emphasizing the responsibility of non-Indigenous staff to champion decolonization and anti-racism work [33, 34].

There are also several other findings that are relevant to the broader healthcare community. For example, another important consideration in the implementation of ICS education programs that arose pertained to finding the physical space that is required for the in-person component of the ICS education program. Although online training can be easier to administer, it is recognized that with this method of delivery it is more difficult to build relationships, which is an important Way of Doing in many Indigenous communities [35]. However, research has shown that utilizing both in-person and online training formats has been beneficial in delivering ICS education programs, with other existing programs also using both asynchronous and synchronous teaching methods to enhance staff’s learning opportunities [15, 21, 28], as was the case in the present study. Additionally, some studies have highlighted that nurses often prefer online training as it is more flexible, self-paced, and generally better suited to their work schedules. However, in-person training provided staff with a more interactive learning experience, and some research has found that some healthcare workers prefer in-person courses due to an already high burden of online training within their roles [15, 21, 28]. Nevertheless, more research into understanding the teaching modalities that are most effective and culturally appropriate is needed, though it is important to recognize that ICS education programs represent only one component of a multi-pronged approach to addressing anti-Indigenous racism within healthcare [16].

In response to the challenges around recruiting staff who may be resistant to ICS education programs, a finding in this study, there have been calls for making attendance compulsory for all healthcare staff [36]. However, some evidence suggests that making ICS education programs mandatory may be ineffective and discourage participants from thoughtfully engaging in the course content and discussions [31, 37]. Therefore, healthcare decision-makers should exercise caution when considering compulsory ICS education programs to avoid diluting the significance and importance of this work, though novel approaches to recruitment – including gifts, food, or other incentives for partaking in ICS education programs – should be considered to encourage participation.

Lastly, grounding ICS evaluation in Indigenous methodologies and Two-Eyed Seeing, such as by in-person sharing circles, reflects Indigenous protocols and helps cultivate a safe place for dialogue, self-reflection, and learning [30, 35, 38]. Participants shared their perspectives on the value of having the opportunity to reflect in this way and the use of Indigenous research and evaluation methodologies should be centered and not overlooked in research. In turn, employing Indigenous methodologies and approaches provided Indigenous participants with an opportunity for deeper reflection on what it meant and how it felt delivering an ICS education program as an Indigenous person, providing a more nuanced understanding of the barriers and facilitators that were unique to Indigenous participants (e.g., the emotional burden of facilitating, the importance of Elder presence, respecting protocols on sharing food, etc.). Despite this, future research seeking to understand Indigenous Peoples’ experiences as leaders in the development and deployment of ICS education programming would be valuable.

There are several limitations to this study. Firstly, this qualitative process evaluation was not longitudinal and therefore did not capture implementation team members perspectives at the beginning nor the end of this pilot project. As a result, this study did not examine implementation team learning outcomes following the completion of the project and may have missed potential challenges or facilitators that arose following the mid-term sharing circle. Additionally, this study was conducted during multiple public health emergencies, including the COVID-19 pandemic, and so findings may not be generalizable to other time periods or settings. Lastly, these findings are specific to a large urban ED in Vancouver, Canada and may not be generalizable to other EDs or healthcare settings.

## Conclusion

There is an ongoing need to expand ICS education programs across all public services, but particularly in healthcare. Despite limited research examining challenges and facilitators to implementing ICS education programs, our study findings provide some insights and considerations when implementing similar programs within other healthcare institutions and settings. Moreover, implementation team members shared the importance of ensuring resources and supports are in place, that Indigenous People lead the design, implementation, and evaluation of ICS education programs, and that aspiring allies are available and willing to do the heavy lifting required to deliver ICS education programs.

## Supplementary Information

Supplementary Material 1.

## Data Availability

The datasets generated and/or analysed during the current study are not publicly available in order to protect participants’ privacy and confidentiality but are available from the corresponding author on reasonable request.
