# Supplementary material for: A qualitative study of challenges and facilitators to implementing an Indigenous-led cultural safety education program within a large urban emergency department in Vancouver, Canada
Source: BMC Med Educ. 2025 Aug 16;25:1167. doi: 10.1186/s12909-025-07736-0 (PMC12357466; doi:10.1186/s12909-025-07736-0)
Supplement: Supplementary file 1 — Supplementary Material 1. [file 12909_2025_7736_MOESM1_ESM.docx]

**Table 1. Preliminary Codebook**

| **Theme** | **Subtheme** | **Definition/Description** | **Example Quotes** |
| --- | --- | --- | --- |
| Challenges | Target program readiness | Comments on the emergency department’s preparedness to host the Indigenous Cultural Safety education program | “Understanding what ICS was, and what was needed, and what was needed for the staff to be ready. I didn't understand that initially. So it was like a knowledge gap for me. And then what was the ask of us and our team to get this training done, was a lot. Like it was - I didn't have that understanding for a while, because [the previous Director] was doing a lot of this [organizing].” |
|  | Staff recruitment | Comments on recruiting emergency department staff to participate in the Indigenous Cultural Safety education program | “And then trying to work out how to put this onto an already taxed staff was really difficult. It’s already been two years of really difficult work. They’ve had enough. How are we going to put this [course] – that's extremely important and coming from a really good place – but we need to get these people, we need to get our people, through it. How do we word that in a way that's going to get the best buy-in possible? And it was really tricky to understand that for a little while until we sat, having a few of these meetings.” |
|  | External events | Comments on events that impacted healthcare system capacity and the delivery of the Indigenous Cultural Safety education program | “There were a lot of challenges that we haven't talked about yet. A global pandemic that put this project on hold for several years, and for good reason. But it did have a significant impact on that planning phase. And that's because we were held off, it can't happen, obviously. Because VGH emerge was a hub of caring for people during this time. And so as a planning team, we were really on hold and then are things getting better, they might be getting a little bit better. We might be ready soon to do this. Oh, nope, there's another wave. No, we're not ready. Okay, now we might be ready again. Nope, we're not ready, false alarm.” |
|  | Time constraints | Comments on time-related pressures on the delivery of the Indigenous Cultural Safety education program | “I think the window of opportunity was really small, coming out of COVID, and then having to prepare for [the new electronic medical record system] to go live, which is very resource intensive.” |
|  | Difficulty securing facilitators | Comments on the challenges faced when recruiting and deploying Indigenous Cultural Safety education program facilitators | “I think that we had challenges with delivery in terms of the comfortability of facilitators. And so what does that training piece look like to be able to deliver the course? And what that translated into was that it was one or two or three people that delivered the course, and there was a lot of urgency around people having to now drop their commitments to be able to go and deliver the course.” |
|  | Changing curriculum | Comments on updates made to the Indigenous Cultural Safety education program curriculum during its roll-out | “I think as a facilitator, one of the challenges was that the curriculum was changing a little bit. Like it was new, right when we launched, so as someone who had facilitated previously, I was like, “OK, it's new, I have to relearn it now”. And then, I think there was a little confusion because one facilitator presented the blanket exercise while another facilitator offered the timeline, and they were meant to be either/or, but then they got overlapped, which then created a time crunch in the workshop, and it also seemed repetitive, so it kind of played with the flow a little bit.” |
|  | Logistical challenges | Comments on the technical challenges to implementing the Indigenous Cultural Safety education program | “It doesn't seem like it was maybe a challenge, but it definitely was, was space for the course. Because that's the whole thing. And I think that needs to definitely be reflected in the research. And if this is a priority, then that space needs to be like made.” |
| Solutions | Project management/coordination support | Comments on project management and coordination support when implementing the Indigenous Cultural Safety education program | “Particularly in terms of recruitment. I don't know if that's planning or where that sort of sits, but I think that would have been a bit more helpful, and maybe that would have come with a project manager on the side? I'm not sure. Or is that like us leading it? I don't know what that looks like. But it was like heavily weighted on [Emergency Department administrators] to do that work, which is hard because you're juggling all the other things.” |
|  | Supporting facilitators | Comments on the supports that can be provided to Indigenous Cultural Safety education program facilitators | “So I think a lot more needs to go into supporting and nurturing and really getting a great group of facilitators who are like ready to be able to deliver. I think that how that resources and the supports are put in place too. Then there's some change and some direction to, and a little mentoring. So I think that, again, that that's something that's going to continue to happen.” |
|  | Supporting the implementation team | Comments on the support that can be provided to Indigenous Cultural Safety education program implementation team | “And then just sort of them knowing that, OK, we have people who are doing it online, but it's not really translating into in-person attendance. How do we add more people in? Can we add more people? So we are reaching out to people, but then their manager’s not understanding what I was asking. And who's going to pay them and those sorts of things, was a lot. And [to the external consultant], true, you actually helped on that as well. But it's just, there are a lot of those sort of difficulties initially, that I sort of had myself. Again, it's the supporting resources, it's just sometimes having those extra people that can actually focus on that a little bit, as well.” |
|  | Flexible scheduling | Comments on Indigenous Cultural Safety education program scheduling to reduce participation barriers for healthcare staff | “And just thinking about this stuff. A lot of nurses are mothers. And so is it possible even have an evening session, which would free their infant childcare along with their days off? Free people to go a little bit more. But just getting that sweet formula, I think, for attendance.” |
|  | Accommodations and incentives | Comments on ways to encourage healthcare staff’s participation in the Indigenous Cultural Safety education program | “I've been to some other trainings that we've had for senior leadership and different groups that were planed specifically for that group. And we've always shared a meal. And that's been really important. Like, so the whole circle happens, and the training happens, and then we share a meal. And it's kind of like the final stamp on the circle. We move away from the circle, we sit, share a meal together, and people get to unwind from what they learned and what they absorbed. And if they were emotional, they get to kind of come down to that next level. And you get to know people more personally. And that's what I felt was maybe missing at this workshop.” |
|  | Cross-departmental communication | Comments on the importance of open, consistent communication between Indigenous Cultural Safety education program implementation team members to support delivery, uptake and overall success | “I think just the only way, I would mitigate maybe some of those planning stage challenges is to just allocate more time to those, that pre implementation phase. Which really just requires a lot of time, relationship building and thoughtful intention around the methods you're going to use to measure the success of the intervention.” |
